# Obstetric analgesia for vaginal birth in contemporary obstetrics: a survey of the practice of obstetricians in Nigeria

**DOI:** 10.1186/1471-2393-14-140

**Published:** 2014-04-12

**Authors:** Lucky O Lawani, Justus N Eze, Okechukwu B Anozie, Chukwuemeka A Iyoke, Nduka N Ekem

**Affiliations:** 1 School of Post Graduate Studies, Department of Community Medicine, University of Nigeria, Enugu, Enugu State, Nigeria; 2 Department of Obstetrics and Gynaecology, Federal Teaching Hospital, Abakaliki, Abalkaliki, Ebonyi State, Nigeria; 3 Department of Obstetrics & Gynecology, University of Nigeria Teaching Hospital, Enugu, Enugu State, Nigeria

**Keywords:** Analgesia, Obstetrics, Practice, Contemporary, Pain

## Abstract

**Background:**

Contemporary obstetrics in sub-Saharan Africa is yet to meet the analgesic needs of most women during child birth for a satisfactory birth experience and expectedly, obstetricians have a major role to play in achieving this.

**Methods:**

This was a questionnaire-based, cross-sectional study of 151 obstetricians and gynecologists that attended the 46th Annual General Meeting and Scientific Conference of the Society of Gynaecology and Obstetrics of Nigeria (SOGON) held in Abakaliki, southeast Nigeria in November, 2012. SOGON is the umbrella body that oversees the obstetric and gynecological practice in Nigeria. Data was collated and analyzed with Epi-info statistical software, and conclusions were drawn by means of simple percentages and inferential statistics using Odds Ratio, with P-value < 0.05 at 95% Confidence Interval (CI) taken to be statistically significant.

**Results:**

Of the 151 participants, males predominated; 110 (72.9%) practiced in government-owned tertiary hospitals in urban locations. Only 74 (49%) offered obstetric analgesia. Among users, only 20 (13.3%) offered obstetric analgesia routinely to parturients, 44 (29.1%) sometimes and 10 (6.6%) on patients’ requests. The commonest analgesia was opioids (41.1%). Among non-users, the commonest reasons adduced were fear of respiratory distress (31.1%), cost (24.7%) and late presentation in labour (15.6%).

**Conclusion:**

The routine prescription and utilization of obstetric analgesia by obstetricians in Nigeria is still low. Obstetricians are encouraged to step up its use to make childbirth a more fulfilling experience for parturients.

## Background

The pain of child birth is arguably the most severe pain most women will endure in their lifetime: it is considered by some societies as a natural phenomenon which should be tolerated [1-3]. Addressing the issue of pain relief during child birth is a way of promoting a satisfactory birth experience and healthy reproductive outcome in women during child bearing [1]. The American College of Gynecologist and Obstetricians (ACOG) recommends that pain management should be provided whenever medically indicated [4]. So also, the National Institute of Clinical Excellence (NICE) of the United Kingdom recommends the education of women on the options and availability of effective analgesia in labour as a means of ensuring that women receive optimal analgesia during child birth [5]. However, the Society of Gynaecology and Obstetrics of Nigeria (SOGON) currently has no clearly stated guidelines for pain management in labour but local studies have shown variation in practice and that women who received obstetric analgesia in form of epidural analgesia had shorter duration of labour, minimal blood loss and were satisfied with their experience of labour than those who did not [6-8]. There have been reports of poor utilisation of analgesia in labour despite high (85.1%) demand for it by Nigerian parturients [8]. In south west Nigeria, 32% of women desired analgesia in labour [9], while in northern Nigeria it was 79.3% of parturients who desired pain relief in labour [6]. In Ibadan, Nigeria it was reported that despite limited availability, women who are aware of epidural analgesia and those who have received it in a previous labour were more likely to want it in their forthcoming labour [10]. These variations in demand could possibly be attributed to ethnicity as reported by some researchers [6,10,11]. It also seems there is a gap between the providers’ attitudes to pain relief in labour and the actual practice of pain relief, such that many providers appear to deny parturients pain relief during child birth without genuine reason(s) [12]. In addition, most parturients in southeast Nigeria has been shown to be unaware of their right to pain relief in labour. Ignorance and fear of unfavorable reactions from caregivers have been cited as the main reasons hindering them from requesting labour analgesia [13]. Denial of pain relief to parturients during labour amounts to some form of violation of their fundamental rights and an unnecessary breach of medical ethics [13].

Since pain relief in labour is an important aspect of the management of pregnant women during child birth [14], efforts to evaluate its practice becomes important in order to determine aspects of it that require improvement. Determining the practice habits of obstetricians is central to these efforts. The aims of this study are to assess if obstetricians practicing in Nigeria provided obstetric analgesia to parturients in labour, the forms of analgesia provided, and the challenges and limitations they faced, with a view to making recommendations for improvement.

## Methods

This was a questionnaire-based, cross-sectional study of obstetricians and gynecologists that attended the 46th Annual General Meeting and Scientific Conference of the Society of Gynaecology and Obstetrics of Nigeria (SOGON) held in Abakaliki, southeast Nigeria in November, 2012. SOGON was established in 1965 as the umbrella body that oversees obstetric and gynaecological practice in Nigeria. All qualified and registered obstetricians-gynecologist in the country are eligible for membership. The SOGON conference is an annual event usually held in the last week of the month of November every year. Ethical clearance for this work was obtained from the Federal Teaching Hospital, Abakaliki (FETHA) research and ethics committee. The questionnaire was pretested on 20 doctors in the surgical department of FETHA, it was thereafter corrected and modified for clarity. It had nine main stems each with multiple close and open-ended questions on participants’ bio-demographic characteristics, use/non-use of obstetric analgesia for vaginal birth only, associated challenges and limitations encountered. The survey considered analgesia for instrumental deliveries and non-instrumental complicated and uncomplicated labour managed by obstetricians in their practice.

The questionnaire was administered to 204 obstetricians and gynecologists that consented to participate in the study but only 151 questionnaires were correctly filled and returned, while 53 questionnaires were either not returned or incorrectly filled, these were excluded from the study. Data was collated and analyzed with Epi-info statistical software version 7.0 (Center for Disease Control and Prevention, USA), and conclusions were drawn by means of simple percentages and Odds Ratio calculated with a 2×2 table, statistical test was by Chi-Square, with P-value < 0.05 at 95% Confidence Interval (CI) taken to be statistically significant.

## Results

A total of 151 questionnaires were analyzed giving a respondent rate of 74.0%. Table 1 shows the bio-demographic characteristics of the participants. A total of 135 (89.4%) were males, and the others (10.6%) females. Their ages ranged from 27 – 59 years, with a mean age (±standard deviation) of 42.0 (±7.4) years. Majority (62 or 41.1%) have been in obstetric practice for 5 years or less, while only seven (4.6%) had practiced for 21 years or more. The mean duration of practice (±sD) was 8.4 (±5.9) years (Table 1). The vast majority; 140 (92.7%) had practices located in urban communities. One hundred and ten (72.9%) practiced solely in government-owned hospitals, while the remainder practiced in private hospital or both. Also, 140 (92.7%) practiced in tertiary hospitals, while the rest practiced in secondary health facilities. None practiced in primary level health institutions.

**Table 1:** Bio-demographic characteristics of participants, N = 151

**Characteristics**	**N (%)**
**Sex**	
Males	135 (89.4)
Females	16 (10.6)
**Age (years)**	
≤30	13 (8.6)
31-40	44 (29.1)
41-50	79 (52.3)
51-60	15 (10.0)
**Duration of practice (years)**	
≤5	62 (41.1)
6-10	33 (21.9)
11-15	39 (25.8)
16-20	10 (6.6)
≥21	7 (4.6)
**Location of practice**	
Urban community	140 (92.7)
Rural community	11 (7.3)
**Hospital of practice**	
Government hospital	110 (72.9)
Both government and private	31 (20.5)
Private	10 (6.6)
**Level of practice**	
Tertiary hospital	140 (92.7)
Secondary hospital	11 (7.3)

A total of 74 (49.0%) participants offered obstetric analgesia to parturients in labour that were either non-assisted or assisted with instruments. Table 2 shows the patterns of use of obstetric analgesia among the 74 participants, forms of obstetric analgesia used, and reasons for non-use of obstetric analgesia by 77 participants. Twenty (13.3%) offered it routinely, while 44 (29.1%) offered it sometimes and 10 (6.6%) on patients’ requests. Opiods were the commonest form, prescribed by 62 (41.1%) participants (Table 2). The opiods used were mainly pethidine and pentazocine hydrochloride. The reasons adduced by 77 participants for not offering obstetric analgesia were fear of respiratory distress, 24 (31.1%), cost 19 (24.7%), and late presentation in labour 12 (15.6%), among others (Table 2). Overall, 131 (86.8%) participants think that obstetric analgesia would offer women a better birth experience (Table 2).

**Table 2:** Pattern of use of obstetric analgesia among obstetricians (N = 74) who provided obstetric analgesia, types of obstetric analgesia offered, reasons for non-use of obstetric analgesia among obstetricians (N = 77) who did not offer obstetric analgesia, and participants opinion on whether obstetric analgesia would offer a better birth experience (N = 151)

**Item**	**N (%)**
**Pattern of use**	**74 (49.0)**
Routinely	20 (13.3)
Sometimes	44 (29.1)
On patient request	10 (6.6)
**Types of obstetric analgesia offered**	
Opioids	62 (41.1)
Psychological support	60 (39.7)
Paracetamol	7 (4.6)
Epidural	3 (2.0)
Entonoux	2 (1.3)
**Reasons for non-use**	**77 (51.0)**
Fear of fetal distress	24 (31.1)
Cost	19 (24.7)
Late presentation in labour	12 (15.6)
Non-availability	7 (9.1)
Fear of prolonged 2nd stage of labour	6 (7.8)
Belief that labour is natural	4 (5.2)
Providers think it’s not necessary	2 (2.6)
Decline by patient	2 (2.6)
Fear of adverse maternal effect	1 (1.3)
**Offer a better birth experience**	**151 (100.0)**
Yes	131 (86.8)
Not sure	12 (7.9)
No	8 (5.3)

The survey inquired about administration of analgesia for instrumental vaginal deliveries and results shows that all those who conducted instrumental deliveries gave obstetric analgesia in form of perineal infiltration of episiotomy area, pudendal nerve and paracervical block with 1% xylocaine solution.

Table 3 shows a comparison of the use/non-use of obstetric analgesia between younger (<50 years) and older (>50 years) participants, as well as between urban and rural practitioners. The comparison between the younger and older age groups was statistically significant with Odds Ratio of 4.37 (1.18-16.18) at 95% CI, P-value = 0.018, while that between urban and rural practitioners was 4.77 (0.99-22.85) at 95% CI, P-value = 0.034 (Table 3).

**Table 3:** Showing comparison of use/non-use of obstetric analgesia between younger (N = 136) and older (N = 15) obstetricians, and obstetricians in urban (N = 140) and rural (N = 11) practice

**Item**	**Use**	**Non-use**	**Proportions**		**OR**	**95% CI**	**χ**^**2**^	**P-value**
	n/N	n/N	users	Non-users				
**Age of obstetrician**								
<50	71/136	65/136	0.52	0.48				
≥50	3/15	12/15	0.20	0.80				
					4.37	1.18-16.18	5.61	0.018
**Location of practice**								
Urban	72/140	68/140	0.51	0.49				
Rural	2/11	9/11	0.18	0.82				
					4.77	0.99-22.85	4.51	0.034

OR = Odd ratio, CI = Confidence interval.

## Discussion

In low income countries like Nigeria, pain relief during labour remains essentially rudimentary [15]. In Nigeria, although parturients perceive labour as a very painful process, studies have shown that only about 22.1% - 33.5% of them receive some form of analgesia [16,17], this is higher than the 18% reported in Kenya but less than 55% in Durban, South Africa [18,19]. This variance between labour pain perception by parturients and the poor provision of analgesia by care givers is therefore worrisome. In addition to the several reasons already adduced for the variance, one question one may ask is if male dominance of obstetric practice in Nigeria has a contributory role. It is noteworthy that 89.4% of the participants in this study were males. There is need for further research to elucidate the extent to which this patriarchy of obstetric profession affects the offer of obstetric analgesia to parturients during labour.

It is pertinent to note that majority of the participants were practicing in tertiary hospitals which are often located in towns and cities out of the reach of majority of the populace who lives in rural communities. Thus, to a large extent, secondary health institutions and the rural communities are often deprived of specialist obstetric care, including but not limited to, provision of analgesia during labour. This disproportionate distribution of specialized obstetric care is not encouraging in a country as populous as Nigeria with a predominantly rural population.

Although majority of the participants had practiced for more than five years and as such are experienced clinicians, yet only 49% of them offered any form of analgesia to parturients during labour. These findings are not encouraging, especially when it is expected that women should be offered among other things, effective pain relief during labour [5]. It however, compares favorably with reports from some tertiary hospitals in Nigeria, like in Zaria where a user rate of 48.8% was reported [12] and 38.9% in Benin, south-south of the country [20] but was much higher than the 22.1% of parturients reported to have received analgesia in labour in Enugu southeast Nigeria [21] and the 11% from the northeast of Nigeria [22]. It is noteworthy that, in this study, only about 13.3% offered pain relief routinely to parturients. With reports of use from tertiary hospitals so low, the situation in many rural hospitals will probably be that no analgesia is available to women in labour [16].

Pain relief is advocated for both natural and induced or augmented labour, more especially in situations of induction/augmentation, since induction or augmentation have been associated with enhanced labour pain which is expected to increase the demand for analgesia by parturients in labour. There were generally limited local studies on the ratio between natural and strictly augmented labour in Nigeria. Available data on augmentation rates are mainly those from neighboring African countries like Benin, Niger, Mali, Ivory Coast, Senegal and Ethiopia [23]. However, studies on induced labour in Nigerian parturients have reported variation in rates between different obstetric units which ranges between 7-13% [23]. This seemingly significant rate will necessitate a higher demand for pain relief in labour in our setting.

There are various techniques of providing obstetric analgesia which may be regional or non-regional. The non-regional forms may be pharmacological or non-pharmacological. In the United Kingdom (UK), the non-regional techniques are employed more often, while in the United States of America, regional analgesia is the most frequently utilized, with the uptake of epidural analgesia as high as 60% [4,5]. In this study, the forms of analgesia offered by participants to parturients during labour and childbirth were limited. Despite their attendant complications, opioids, offered by 41.1% of the participants, were the commonest form of pharmacological analgesia utilized. This may be because opioids are readily available and do not require special skills to administer. Opioids, however, have limited pain relief effect [20]. Other forms of pharmacological analgesia, such as paracetamol and entonoux, were used only by a handful of the participants. Surprisingly, as many as 39.7% of the participants offered psychological support, a form of non-pharmacological analgesia, even when there are no consistent evidence that this technique affects labour pain [3]. Regrettably, the trending epidural analgesia was employed only by a paltry 2% of the participants. This is despite the fact that studies have shown that inhalational method and intravenous administration of opioids for pain relief in labour are fast giving way to epidural analgesia [20], that epidural analgesia is acceptable to women in our setting, with no increase in adverse outcome [7], and that those who had received it in previous labour where more likely to request it in their subsequent labour [17].

According to the participants that did not offer pain relief to their parturients, their actions were advised by the fear of fetal distress from respiratory depression, the cost of the medications, and the fact that parturients presented very late in labour, among others (Table 2). Opioid are the most widely used analgesia in labour in Nigeria as seen in this study, and the fear of possible respiratory depression was mainly due to its depressive side effect on the respiratory center, especially when administered in the later stages of labour or within 4 hours of delivery. The reasons for non administration of analgesia in labour by non users corroborates with the results of other studies from Nigeria [5,9,21,24]. It is however, noteworthy that unrelieved labour pains may have untoward effects on maternal and fetal health. Labour pain is associated with reflex increase in blood pressure, oxygen consumption, and liberation of catecholamines, all of which could adversely affect uterine blood flow. A long, painful labour may leave a mother exhausted, frightened, hysterical and even incapable of making decisions [15]. Severe labour pains could result in mental health disturbances which may interfere with maternal–neonatal bonding and future sexual relationships. It may also contribute to postpartum depression and rarely, to post traumatic stress disorder [20,25]. This goes to support the fact that obstetricians through the practice of routinely offering obstetric analgesia can significantly improve the maternal and perinatal outcomes of pregnancy. Hence, healthcare professionals are enjoined to consider how their own values and beliefs inform their attitude to coping with pain in labour and ensure their care supports the woman’s choice [5]. The results from this study also shows a major trend between the practice of younger and older obstetricians in offering pain relief to parturients in labour, as well as trends between rural and urban practice, this may be an indication that some future changes might be expected in the practice of routinely offering parturients pain relief in labour.

### Limitation of the study

This study surveyed the practice of obstetricians in Nigeria, considering that most birth in the country occur outside the facilities of practice of most obstetricians, seeking the opinion of midwives, non-specialist medical practitioners and traditional birth attendants who supervises and conduct most deliveries in the country would have enhanced the findings of this work. Despite a high response rate, the findings may not be a total reflection of the practice of all obstetricians in the different regions of the country, since not all members of SOGON participated in the study.

## Conclusions

Pain relief during labour is desired by many women and contributes immensely to their satisfaction of the experience of childbirth. Unrelieved, labour pains may impact negatively on the lives of parturients to such an extent that her baby and family may also be affected. Unfortunately, obstetricians practicing in Nigeria do not yet routinely provide pain relief to all parturients in labour in keeping with international recommendations.

We recommend that SOGON, the Federal Ministry of Health, and health policy makers in Nigeria should develop a national protocol on obstetric analgesia for obstetric caregivers. The protocol should be evidence-based and within the limits of available manpower, resources and technology in Nigeria. It should be matched with training and retraining of obstetric caregivers to improve their acceptance of and proficiency in the available forms of obstetric analgesia in the country. Efforts should also be made to address the participants’ reasons for non-provision of obstetric analgesia, as elucidated by this study. There is need for team work by all the stakeholders in the health sector and the government to achieve these. There is also need for ongoing research and appraisal of the forms and use of obstetric analgesia in the country with a view to ensuring that it is not only readily available, but that ultimately, the standard achieved meets the internationally accepted standard.

## Competing interests

The authors declare that they have no competing interests.

## Authors’ contributions

LOL designed the study, oversaw its conduct, data analysis, and interpretation and drafted the original article and reviewed the final draft. JNE contributed to the design of the study, collection and analysis of the data, and review of the article. OBA assisted in the drafting of the article, data analysis, interpretation and review of the article. CAI participated in the design of the study, collection, analysis and interpretation of data as well as the review of the final manuscript. NNE was involved in the analysis of the results and drafting of the original article. All the authors read and approved the final draft of the article.

## Pre-publication history

The pre-publication history for this paper can be accessed here:

http://www.biomedcentral.com/1471-2393/14/140/prepub

## Study Area

This study was conducted at the Society of Gynecology and Obstetrics of Nigeria (SOGON) 46th annual conference hosted by the Federal Teaching Hospital Abakaliki (FETHA).
